# Effect of Sodium Lauryl Sulfate-Fumaric Acid Coupled Addition on the *In Vitro* Rumen Fermentation with Special Regard to Methanogenesis

**DOI:** 10.4061/2010/858474

**Published:** 2009-10-29

**Authors:** M. A. Abdl-Rahman, F. A. R. Sawiress, A. M. Abd El-Aty

**Affiliations:** ^1^Department of Physiology, Faculty of Veterinary Medicine, Cairo University, Giza 12211, Egypt; ^2^Department of Pharmacology, Faculty of Veterinary Medicine, Cairo University, Giza 12211, Egypt

## Abstract

The aim of the current study was to evaluate the effect of sodium lauryl sulfate-fumaric acid coupled addition on *in vitro* methangenesis and rumen fermentation. Evaluation was carried out using *in vitro* gas production technique. Ruminal contents were collected from five steers immediately after slaughtering and used for preparation of inoculums of mixed rumen microorganisms. Rumen fluid was then mixed with the basal diet of steers and used to generate four treatments, negative control (no additives), sodium lauryl sulfate (SLS) treated, fumaric acid treated, and SLS-fumaric acid coupled addition treated. The results revealed that, relative to control, efficiency in reduction of methanogenesis was as follows: coupled addition > SLS-addition > fumaric acid addition. Both SLS-addition and SLS-fumaric acid coupled addition demonstrated a decremental effect on ammonia nitrogen (NH_3_–N), total short chain volatile fatty acids (SCVFAs) concentrations and the amount of substrate degraded, and an increment effect on microbial mass and microbial yield (Y_ATP_). Nevertheless, fumaric acid did not alter any of the previously mentioned parameters but induced a decremental effect on NH_3_–N. Furthermore, both fumaric acid and SLS-fumaric acid coupled addition increased propionate at the expense of acetate and butyrate, while, defaunation increased acetate at the expense of propionate and butyrate. The pH value was decreased by all treatments relative to control, while, cellulase activity did not differ by different treatments. The current study can be promising strategies for suppressing ruminal methane emissions and improving ruminants feed efficiency.

## 1. Introduction

Ruminal methanogenesis represents a loss of feed energy for ruminants and a significant source of global warming and pollution into our atmosphere. Energy lost as enteric methane from mature cattle ranges from 2–12% of gross energy intake [1] depending on diet composition [2–4]. Inhibition of methanogenesis may therefore have significant economical and environmental benefits [5].

 Many feed additives have been developed to improve the efficiency of nutrient use by decreasing the total amount of methane production, among which ionophore antibiotics have been very successful [6]. However, the chance to find antibiotics residues in milk and meat and their effects on human health suggests to look closer to decrease their use and seek for safer alternatives.

 Generally, methane is produced by two types of methanogens, the slow growing methanogens (generation time 130 hours) that produces methane from acetic acid, and fast growing methanogens (generation time 4–12 hours) that reduce CO_2_ with H_2_. In the rumen, methanogenesis occurs mostly by the later pathway as ruminal retention times are too short to permit establishment of the slow growing species [7].

Newbold et al. [8] had described an intimate metabolic correlation between methanogenic bacteria and ciliate protozoa. Protozoa consume oxygen [9] and oxygen levels were found to increase transiently in defaunated animals that adversely affect methangenic archea [10].

Theoretically, methanogenesis can be reduced by decreasing H_2_ production or by increasing H_2_ utilization. However, direct inhibition of reactions that form H_2_ may depress fermentation in microorganisms producing H_2_, including the main cellulolytic bacteria, because H_2_ production is a mean for the disposal of electrons liberated by the oxidation of energy-yielding substrates [11]. On the other hand, increasing H_2_ utilization by organisms other than methanogens requires addition of an appropriate electron acceptor and an efficient type of rumen bacteria that can perfectly utilize such acceptor in production of a more beneficial product, namely, propionate. These include fumarate utilizing bacteria (*Bacteroides ruminicola*, *Bacteroides succinogens* and *Selenomonus ruminantium*) [12–14].

 Propionate precursors (malate, fumarate, and succinate) were found to be a desirable alternative route for hydrogen disposal leading to reduction of methanogenesis and enhancement of propionate production [12–14]. However the affinity of fumarate reducing bacteria to hydrogen was lower than the affinity of methanogens, so methanogenic microorganisms can outcompete fumarate reducing bacteria at low hydrogen concentration normally present in the rumen [12].

 On the contrary, defaunating agents (e.g., Sodium lauryl sulphate) were found to strongly inhibit methanogenesis [15]. However accumulation of hydrogen would prevent further degradation of organic matter [16].

 Could Lauryl sulfate-fumaric acid coupled addition overcome microbiological constraints of single feed additive-supplementation and achieve sustained inhibition of rumen methanogenesis? This was the aim of this investigation.

## 2. Materials and Methods

This work was carried out in the Department of Physiology, Faculty of Veterinary Medicine, Cairo University, Egypt. The experimental protocol was approved by the Institutional Animal Care and Use Committee of the Faculty of Veterinary Medicine, Cairo University.

### 2.1. Collection of Rumen Contents

Ruminal contents used to prepare the treatment systems were collected from the rumen of five slaughtered steers and immediately transferred to the laboratory in a prewarmed thermos flask. Collected ruminal fluids were handled as described by Callaway and Martin (1997) [17]. Ruminal fluids were strained through four layers of cheesecloth into a separating flask previously gassed with oxygen-free CO_2_ and brought immediately to the laboratory. The samples were then incubated under anaerobic conditions at 39°C for up to 60 minutes to allow small feed particles to buoy up and the microbial fractions to sediment at the bottom. Small feed particles that had floated to the surface were removed, and the particle-free fluids were mixed with the buffer solution of Goering and Van Soest [18] in the proportion 1 : 2 (v/v), flushed with oxygen-free CO_2_, and used as inoculums of mixed rumen microorganisms.

### 2.2. Preparation of Treatment Systems and In Vitro Fermentation

The method used for *in vitro *fermentation was based on the technique described earlier by Menke et al. [19], and slightly modified by Ngamsaeng and Wanapat [20]. Two- hundred milligrams of feed sample (composition and chemical analysis are shown in Table 1) were weighed into 60 mL calibrated plastic syringes with pistons lubricated with vaseline. Approximately 30 mL of buffered rumen fluid was dispensed into the syringes and the following treatment systems were then prepared for each sample in a duplicate syringes per treatment: negative control (no additives), sodium lauryl sulfate treated (0.01 mg/mL), fumaric acid treated (0.5 mg/mL), and sodium lauryl sulfate-fumaric acid coupled treated (0.01 and 0.5 mg/mL of each), respectively. After gentle shaking, syringes were incubated at 39°C and the volume of gas produced was recorded at 24 hours post incubation. For each sample, duplicate syringes per treatment were incubated to be used for measurement of *in vitro* true degradability with concomitant microbial mass generated.

### 2.3. Analysis

#### 2.3.1. Determination of pH

After termination of incubation, the fluid samples were drawn into plastic bottles and pH was immediately determined using pH meter.

#### 2.3.2. Determination of Total VFAs Concentrations and Individual VFAs Proportions

For determination of total VFAs concentrations and individual VFAs proportions 1 mL of 25% meta-phosphoric acid was added to 5 mL of fermentation fluids, centrifuged (7 000× g for 10 minute) and supernatants were stored at −20°C until analyzed. Total VFAs concentrations were measured by steam distillation [21] and molar proportions of VFAs were analyzed using High Performance Liquid Chromatography (HPLC; Model Water 600; UV detector, Millipore Crop.) according to the method of Mathew et al. [22].

#### 2.3.3. Determination of Ammonia Nitrogen Concentration

Two mL sample of fermented fluid was acidified with 2 mL of 0.2 *N* HCl and frozen. Samples were centrifuged at 5000× g for 20 minute, and the supernatant was analyzed by spectrophotometry [23] for ammonia N.

#### 2.3.4. Calculation of Fermentative CO_2_ and CH_4_

Fermentative CO_2_ and CH_4_ in the buffered rumen fluid were estimated by the equations of Wolin [24], which have been validated recently by Blümmel et al. [25] as following:

(1) $$\text{Fermentative }{\text{CO}}_{2}=\text{A}/2+\text{P}/4+1.5\text{B}$$

where A, P and B are moles of acetate, propionate, and butyrate, respectively.

(2) $$\text{Fermentative C}{\text{H}}_{4}=\left(\text{A}+2\text{B}\right)-{\text{CO}}_{2}$$

where A and B are moles of acetate and butyrate respectively and CO_2_ is moles of CO_2_ calculated from previous equation.

#### 2.3.5. Measurement of Extracellular Cellulase Activity

Supernatant from each fermentation fluid sample was separated by centrifugation at 3 000 rpm for 20 minute. Half mL of the supernatant (crude enzyme solution) was mixed with 0.5 mL of 1% carboxymethyl cellulose (CMC) solution in 0.05 M sodium citrate buffer. The reaction proceeded for 1 hour at 55°C without shaking, and then stopped by boiling for 5 minute. Boiled samples were centrifuged at 7 000 rpm for 5 minute, and reducing sugars produced in the supernatants was measured colorimetrically [26]. One unit of enzyme activity was defined as the amount of enzyme that produced 1 mmoL of glucose equivalent of reducing sugar per minute.

#### 2.3.6. Measurement of In Vitro True Degradability with Concomitant Microbial Mass Generated

*In vitro* true degradability was determined according to the procedures of Blümmel et al. [27]. The remaining contents of the separate syringes were drained into beakers and syringes were thoroughly washed with neutral detergent solution (NDS). The contents were digested with NDS for one hour to solubilize microbes and obtain only the undegraded feed. The contents were then filtered, dried at 130°C for 2 hour, and weighed. True degradability was then calculated as the weight of substrate incubated minus the weight of the residue after NDS treatment. The microbial mass generated by termination of incubation could then be estimated according to Grings et al. [28] as follows:

(3) $$\begin{array}{cc} \text{microbial mass}\;\left(\text{mg}\right) & =\text{mg}\;\text{ substrate truly degraded}\\ & \;-\left(\text{mL gas volume}\times 2.2\right), \end{array}$$

where 2.2 is a stoichiometrical factor. 

The microbial yield (Y_ATP_) was then calculated as the mg microbial mass produced per mmole ATP generated in fermentation of carbohydrates to VFAs. The moles of ATP generated per mole of SCVFAs are 2 for acetate, 3 for propionate, 3 for butyrate, and 1 for methane [29].

### 2.4. Statistical Analysis

Data were analyzed by one way analysis of variance (ANOVA) test according to Snedecor and Cochran [30]. Treatment means were compared by the least significance difference (LSD) at 5% level of probability.

## 3. Results

Table 2 identifies that total gas production was decreased by defaunation and by SLS-fumaric acid coupled addition, but it was not affected by fumaric acid addition. However, the decremental effect induced by coupled addition surpassed the decremental effect of defaunation (32.7% versus 18.3%). Furthermore, the means of different treatment systems denote that SLS-fumaric acid coupled addition had a tremendous damping effect on CO_2_ and CH_4_ production (40.1% and 43.07%, resp.). In contrast, CO_2_ and CH_4_ were moderately reduced by defaunation (21.6% and 17.7%, resp.). However, fumaric acid addition did not alter the output of CO_2_ but induced a slight reduction in CH_4_ (11%). 

Data presented in Table 3 reveals that, the pH value of the fermentation fluid was decreased by all treatments relative to control, however decremental effect of both fumaric acid addition and SLS-fumaric acid coupled addition exceed that recorded by defaunation. Meanwhile, ammonia nitrogen concentration and total SCVFAs were decreased by both defaunation and SLS-fumaric acid coupled addition; however, the decremental effect induced by coupled addition outdid that induced by defaunation (57% versus 47.3 % for ammonia nitrogen and 36.3% versus 20.4% for total SCVFAs). In contrast, addition of fumaric acid did not alter the concentration of total SCVFAs but induced a decremental effect on ammonia nitrogen concentration. Furthermore, the overall mean of VFAs molar proportions reveals that both fumaric acid addition and SLS-fumaric acid coupled addition were associated with increased propionates at the expense of acetate and butyrates and therefore, a lowered A/P ratio was recorded for both treatments. Conversely, defaunation was associated with increased acetate at the expense of propionates and butyrates so, recorded a higher A/P ratio.

It is evident from Table 4 that the extracellular cellulase activity within the fermentation fluid did not alter by different treatment systems relative to control. Nevertheless, the amount of substrate degraded was reduced by both defaunation and SLS-fumaric acid coupled addition, while it was not affected by the addition of fumaric acid. In contrast, microbial mass generated during fermentation was increased by both defaunation and SLS-fumaric acid coupled addition while, it also was not affected by addition of fumaric acid. Additionally, microbial yield/mmole ATP generated during fermentation (Y_ATP_) was increased by both defaunation and SLS-fumaric acid coupled addition, however, the increment effect of SLS-fumaric acid coupled addition was overwhelming (84% versus 36.5% achieved by defaunation). Nevertheless, fumaric acid addition did not alter Y_ATP_.

## 4. Discussion

*In vitro* gas production technique has been proved to be a potentially useful and less expensive technique for feed evaluation in developing countries [27, 31, 32]. To our knowledge this is the first *in vitro* study conducted to assess the effect of this coupled addition on rumen methane production.

Because several electron acceptors were found successful in diverting H_2_ away from methanogens (fumarate was the most effective candidate), authors thought that research efforts should be focused on enhancing the activity of fumarate-utilizing bacteria and in this regard defaunation was tested as a tool for potentiating the activity of fumarate-utilizing bacteria and enhancing the capacity of ruminal ecosystem to reduce fumarates into propionates. 

From the foregoing results it has been observed that coupling defaunation with fumaric acid addition achieved an overwhelming decrease in total gas volume, methane and CO_2_ production (32.7%, 43.07%, and 40.1%, resp.). The 17.7% reduction in CH_4_ production achieved by defaunation could be really attributed to the intimate metabolic correlation existing between methanogenic microorganisms and ciliate protozoa so that defaunation reduced methanogenesis and directed hydrogen for other metabolic processes presumably acetate and propionate production [33, 34], however, the response was lower than that recorded by Ushida et al. [35] and Santra et al. [15]. On the other hand, the 11% decrease in CH_4_ production recorded by fumaric acid addition was lower than that recorded by López et al. [13] and Bayaru et al. [14] but was higher than that recorded by Carro and Rannila [36]. These differences could probably be attributed to a diet and dose related factors. It appears that the maximum potential of fumarate to divert H_2_ away from CH_4_ is limited presumably because methanogens utilize H_2_ more rapidly than fumarate-utilizing bacteria. Asanuma et al. [12] suggested that fumarate-utilizing bacteria have a disadvantage in the utilization of H_2_ compared with methanogens when the partial pressure of H_2_ is low. However, addition of a defaunating agent (SLS) attenuated methanogenic microorganisms and potentiated fumarate utilizing bacteria, making them able to override methanogens and methanogenesis was greatly reduced.

### 4.1. Effect of Defaunation, Fumaric Acid Addition and SLS-Fumaric Acid Coupled Addition on Extracellular Cellulase Activity

Because one of the greatest merits of ruminants is the ability to utilize fiber, methane production should be reduced without depressing fiber digestion. Extracellular cellulase activity within the fermentation fluid was not altered by different treatment systems relative to control which indicates efficient H_2_ disposal without negative drawbacks on cellulolytic bacterial activity. Nevertheless, reduction of substrate degradability by both defaunation and SLS-fumaric acid coupled addition may be related to loss of the stabilizing role that protozoa have on the physicochemical characteristics of the ruminal environment [37] and in this regard our results are in accordance with Dohme et al. [38] and Koeing et al. [39].

### 4.2. Effect of Defaunation, Fumaric Acid Addition and SLS-Fumaric Acid Coupled, Addition on In Vitro Fermentation Pattern, Microbial Yield and Substrate Degradability

The pH values in the present incubations were much higher than the critical value of 6.0 enabling maximal CH_4_ formation [40]. The observed increases in propionate production by both fumaric acid addition and SLS-fumaric acid coupled addition could stem from fumarate fermentation itself. The recorded decrease in CH_4_ corresponded well to the issue that hydrogen diverted away from methane was used to reduce and convert fumarate into propionate with little production of acetate and butyrate. This confirms the possibility that these fumarate-utilizing bacteria compete with methanogens for the utilization of H_2_. Additionally protozoa ingest both starch grains and amylolytic bacteria associated with them [41]. These amylolytic bacteria are succinate producing and act synergistically with succinate decarboxylating *Selenomonas ruminantium* to give propionates leading to better energy use since propionate metabolism is more favorable than acetate and butyrate ones [42]. More puzzling is the high yield of acetate at the expense of propionates and butyrates associated with defaunation. The reason for this is not clear but some guesses might be made. First of all, the conversion of glucose to acetate, propionate and butyrate in the rumen results in an overall net release of a reducing power and much of this is used by methanogenic microorganisms to reduce CO_2_ to CH_4_, similarly hydrogen can also be used by acetogenic bacteria in reduction of CO_2_ to acetate (reductive acetogenesis). One of the major constraints to induction of the acetogenic pathway is that the hydrogen threshold for acetogenesis is higher than for methanogenesis as shown by Joblin [43] who concluded that acetogenesis may find its ruminal application when methanogenesis is inhibited. Therefore, high acetate accompanying defaunation in this *in vitro* study might be the end result of enhanced acetogenesis after suppression of protozoal support to methanogenic microorganisms and/or protozoal predation against fibrolytic bacteria is inhibited by defaunation. 

The tendency to lower NH3-N and total SCVFAs concentrations in the fermentation fluid observed by defaunation and coupled addition could be due to a greater utilization by rumen microbes as both microbial mass and microbial yield (Y_ATP_) were increased in accordance to the decremental effect of each of the previous treatments. Leng [44] and Blümmel et al. [45] hypothesized that an inverse relationship exists between gas or SCVFA production and microbial biomass yield. Additionally the overwhelming microbial cell yield achieved by coupled addition (84% versus 36.5% achieved by defaunation) is probably related to the possession of a system for the regeneration of ATP by electron transport phosphorylation coupled with propionate producing bacteria but absent in acetate producing bacteria [46], which implies that these bacteria acquire energy from H_2_ and which otherwise are used to produce methane as suggested by Asanuma et al. [12]. Furthermore, reduction of methane production by coupled addition (43.07%) was more efficient and outperformed methane reduction by defaunation (17.7) and this probably saved more energy as reported by Johnson et al. [47] to meet the synthetic needs of rumen microbes that require a synchronous supply of energy and NH3-N as suggested by Huber and Herrara [48]. 

In conclusion, sodium lauryl sulfate-fumaric acid coupled addition could achieve an overall complementary effect on reduced methanogenesis which was reflected positively on propionates production, ATP production and microbial mass yield. However, further long-term *in vivo* studies are required before putting it to practical use.

**Conflicts of Interest**

The authors declare that there are no conflicts of interest.

**Funding**

This research received no specific grant from any funding agency in the public, commercial or not-for-profit sectors.

## Figures and Tables

**Table 1:** Composition and chemical analysis of the used basal diet.

Ingredients	%
Barely, grain	39.56
Berseem hay	40.00
Wheat straw	20.14
Vitamin and mineral premix	0.30

Chemical analysis	% of dry matter

Crude fibers	31
Crude proteins	13
Ether extract	2.8
Nitrogen free extract	32.5
total ash	10.6

**Table 2:** Effect of treatment systems on gas production and output of CO_2_ and CH_4_ by mixed rumen microorganisms after 24 hours.

Parameter	Control (no additives)	SLS-addition (defaunation)	Fumaric acid addition	SLS-Fumaric acid coupled addition	L.S.D.
Gas volume (mL)	25.10^(a)^ ± 0.83	20.50^(ab)^ ± 0.42	25.20^(b)^ ± 1.24	16.90^(ab)^ ± 0.43	2.41
CO_2_ (*μ*moL)	318.4^(a)^ ± 11.70	249.6^(ab)^ ± 5.88	301.2^(b)^ ± 17.13	190.6^(ab)^ ± 6.56	33.76
CH_4_ (*μ*moL)	144.4^(a)^ ± 5.36	118.8^(ab)^ ± 3.65	128.4^(ac)^ ± 8.64	82.2^(abc)^ ± 3.09	15.84

Data presented as means ± SE, *N* = 5

Values having the same letter in the same raw are significantly different at *P* < .05.

**Table 3:** Effect of treatment systems on fermentation pattern by mixed rumen microorganisms after 24 hours *in vitro* incubation.

Parameter	Control (no additives)	SLS- addition (defaunation)	Fumaric acid addition	SLS-Fumaric acid coupled addition	L.S.D.
pH value	6.95^(a)^ ± 0.0051	6.88^(ab)^ ± 0.0140	6.79^(ab)^ ± 0.0037	6.76^(ac)^ ± 0.0160	0.0332
Ammonia N. conc. (mg/dL)	21.44^(a)^ ± 0.18	11.29^(ab)^ ± 0.72	10.15^(ac)^ ± 0.48	9.20^(ab)^ ± 0.10	1.333
Total SCVFAs conc. (*μ*moL)	542.4^(a)^ ± 19.86	431.6^(ab)^ ± 10.05	544.6^(b)^ ± 29.96	345.4^(ab)^ ± 10.26	58.02
Acetic acid (moL/100 moL)	51.44^(a)^ ± 0.05	54.08^(ab)^ ± 0.36	49.93^(abc)^ ± 0.36	50.0^(abd)^ ± 0.48	1.05
Propionic acid (moL/100 moL)	30.49^(a)^ ± 0.017	27.75^(ab)^ ± 0.006	34.10^(abc)^ ± 0.016	33.84^(abd)^ ± 0.160	0.244
Butyric acid (moL/100 moL)	16.78^(a)^ ± 0.022	16.36^(ab)^ ± 0.013	14.55^(abc)^ ± 0.016	14.73^(abc)^ ± 0.017	0.053
Acetic/propionic ratio	1.69^(a)^ ± 0.010	1.95^(ab)^ ± 0.013	1.46^(abc)^ ± 0.010	1.48^(abd)^ ± 0.015	0.037

Data presented as means ± SE, *N* = 5.

Values having the same letter in the same raw are significantly different at *P* < .05.

**Table 4:** Effect of treatment systems on extracellular cellulase activity, amount of substrate truly degraded, microbial mass, and biomass yield after 24 hours *in vitro* incubation.

Parameter	Control (no additives)	SLS-addition (defaunation)	Fumaric acid addition	SLS-Fumaric acid coupled addition	L.S.D.
Extracellular cellulase activity (mmoL of glucose equivalent/min)	4.30 ± 0.11	4.27 ± 0.07	4.29 ± 0.05	4.28 ± 0.07	NS
Amount of substrate truly degraded (mg)	78.73^(a)^ ± 1.33	71.39^(ab)^ ± 0.66	78.94^(b)^ ± 1.98	65.63^(ab)^ ± 0.69	3.853
Microbial mass generated (mg)	23.51^(a)^ ± 0.49	26.29^(ab)^ ± 0.26	23.50^(b)^ ± 0.75	28.45 ± 0.26	1.450
Microbial yield/mmoL ATP generated (Y_ATP_)	16.80^(a)^ ± 1.14	22.93^(ab)^ ± 0.72	16.33^(b)^ ± 1.52	30.92^(ab)^ ± 1.32	3.632

Data presented as means ± SE, *N* = 5.

Values having the same letter in the same raw are significantly different at *P* < .05.
